# Female Gender Is a Social Determinant of Diabetes in the Caribbean: A Systematic Review and Meta-Analysis

**DOI:** 10.1371/journal.pone.0126799

**Published:** 2015-05-21

**Authors:** Natasha Sobers-Grannum, Madhuvanti M. Murphy, Anders Nielsen, Cornelia Guell, T. Alafia Samuels, Lisa Bishop, Nigel Unwin

**Affiliations:** 1 Faculty of Medical Sciences, University of the West Indies, Bridgetown, Barbados; 2 MRC Epidemiology Unit and UKCRC Centre for Diet and Activity Research (CEDAR), University of Cambridge, Cambridge, United Kingdom; 3 Chronic Disease Research Centre, Tropical Medicine Research Institute, University of the West Indies, Bridgetown, Barbados; Mayo Clinic, UNITED STATES

## Abstract

**Background:**

Diabetes (DM) is estimated to affect 10–15% of the adult population in the Caribbean. Preventive efforts require population wide measures to address its social determinants. We undertook a systematic review to determine current knowledge about the social distribution of diabetes, its risk factors and major complications in the Caribbean. This paper describes our findings on the distribution by gender.

**Methods:**

We searched Medline, Embase and five databases through the Virtual Health Library, for Caribbean studies published between 2007 and 2013 that described the distribution by gender for: known risk factors for Type 2 DM, prevalence of DM, and DM control or complications. PRISMA guidance on reporting systematic reviews on health equity was followed. Only quantitative studies (n>50) were included; each was assessed for risk of bias. Meta-analyses were performed, where appropriate, on studies with a low or medium risk of bias, using random effects models.

**Results:**

We found 50 articles from 27 studies, yielding 118 relationships between gender and the outcomes. Women were more likely to have DM, obesity, be less physically active but less likely to smoke. In meta-analyses of good quality population-based studies odds ratios for women vs. men for DM, obesity and smoking were: 1.65 (95% CI 1.43, 1.91), 3.10 (2.43, 3.94), and 0.24 (0.17, 0.34). Three studies found men more likely to have better glycaemic control but only one achieved statistical significance.

**Conclusion and Implications:**

Female gender is a determinant of DM prevalence in the Caribbean. In the vast majority of world regions women are at a similar or lower risk of type 2 diabetes than men, even when obesity is higher in women. Caribbean female excess of diabetes may be due to a much greater excess of risk factors in women, especially obesity. These findings have major implications for preventive policies and research.

## Introduction

The World Health Organization’s Commission on the Social Determinants of Health(CSDH)[1] drew attention to the marked inequalities in health that exist between and within populations, and focussed attention on health inequities. One of the approaches taken by the CSDH was to examine ‘priority public health conditions’, such as tuberculosis, neglected tropical diseases, mental health, cardiovascular diseases and diabetes[2]. The overall aim of these condition specific analyses was to understand the impact of social determinants on their occurrence and consequences and the potential for public health programmes to reduce health inequities.

Diabetes (DM) is estimated to affect between 10% -15% of the adult population in the Caribbean region[3], and to be a major contributor to premature mortality [3, 4].Where studies have been done, DM is responsible for high rates of complications, such as lower limb amputation [5]. In other parts of the world it is well known that the incidence and prevalence of Type 2 DM, which accounts for over 90% of DM in most populations, tends to be unevenly distributed by some markers of socio-economic position (SEP), including education and income [6]. In high income countries, for example, prevalence and incidence of type 2 DM is higher in those with lower education and income [6]. The relationship between type 2 DM and gender is less clear. Globally, for example, the prevalence in men and women is very similar [3, 7]. More recently, however, it has been reported that once major risk factors for type 2 DM are taken into account, in particular obesity, men have a higher incidence than women [8]. Understanding how diabetes is distributed by gender and what factors underlie that distribution is important for planning interventions to control and prevent this common condition.

In the work described here we sought to determine what is currently known about health inequities and their determinants for DM in the Caribbean with a view to informing health policy and further research. We used the theoretical framework from the CSDH analyses of Priority Public Health Conditions [2], which has five levels and three dimensions, as shown in the figure in the protocol (S1 Appendix). Our specific aim was to determine what is currently known about the social distribution of DM (type 1 and type 2); the social distribution of the risk factors for type 2 DM (‘vulnerabilities’ in the framework); and the social distribution of its major complications (‘consequences’), including DM related mortality, in the Caribbean. As described in the methods section, ‘social distribution’ was interpreted to mean the distribution by the following markers of socio-economic position: gender, race/ethnicity, education, occupation and income. Available data from studies conducted in the Caribbean on all of these, with the exception of gender, was very limited. In this paper, therefore, we present the findings on the distribution by gender of risk factors for type 2 diabetes, diabetes, and diabetes control and complications.

## Methods

### Protocol and eligibility criteria

A study protocol was developed in advance (Supporting Information: S1 Appendix). The final protocol for this review was informed by an initial scoping exercise undertaken in Pubmed and Embase to gain insight into the volume, timeliness and range of published literature available to meet the aim.

We sought literature from studies conducted in the Caribbean that described the distribution by one or more markers of socio-economic position on one or more of the following outcomes:

1. Established risk factors for type 2 DM:
  - Generalised (e.g. mean body mass index or proportions obese) and central obesity (e.g. mean waist circumference or proportions with abdominal obesity);
  - Physical inactivity;
  - Tobacco smoking;
  - Measure of quality of diet (e.g. consumption of fresh fruits and vegetables, or diet quality score);
  - Metabolic syndrome
2. The incidence or prevalence of type 1 and type 2 DM, including reported diagnosis by a health professional and/or based on glucose or glycated haemoglobin measurement
3. In those with diabetes, assessment of disease ‘control’ based on one or more of glycated haemoglobin, fasting glucose and blood pressure.
4. In those with diabetes, mortality and/or the incidence or prevalence of retinopathy, nephropathy, neuropathy, diabetic foot, lower extremity amputation.

We were guided in our choice of markers of socio-economic position by the Progress-plus check list[9] and by the markers predominantly used by the Commission on the Social Determinants of Health [1]. We focussed on identifying data on distribution of risk factors, disease, control or complications by: gender, ethnicity/race, education, income, occupation.

We defined ‘the Caribbean’ as consisting of 28 countries and territories [10]. For cultural and historical reasons three mainland countries are considered part of the Caribbean. These are Belize, Guyana and Suriname, and each is a member of Caribbean Community (CARICOM). Bermuda, which is not geographically part of the Caribbean, was also included as it is an associate member of CARICOM, sharing cultural and historical ties with the English-speaking Caribbean. We did not apply language limits to our search.

We deliberately kept eligibility criteria broad, wishing to identify any study that presented data on the distribution of one or more of the outcomes of interest by at least one of the markers of socio-economic position.

Inclusion Criteria:

- Persons living in the Caribbean;
- Age of study participants: for diabetes outcomes, only studies in adults (18 and older) were considered, whereas for risk factors studies that included children/adolescents (12 years and older) were also included;
- Described the distribution of at least one of the outcomes of interest by one of the markers of socio-economic position;
- Quantitative study design (observation or intervention) with minimum sample size of 50.

Exclusion Criteria:

- Narrative review papers, commentaries, case series, qualitative studies and single case reports were not included.
- Studies that only included the Caribbean diaspora (as opposed to populations living within the Caribbean) were not included.

We limited our final search to studies published between January 1^st^ 2007 and December 31^st^ 2013, with the intention of identifying data that are timely enough to be able to inform policy and further research around the control of DM and related NCDs within the region.

### Information sources, search strategy and study selection

We searched Medline (through Pubmed) and Embase (through Ovid), plus an additional five data bases through the Virtual Health Library (VHL)[11]. The databases searched through the VHL are: LILACS (the database of the Latin American and Caribbean of Health Sciences Information System); MedCarib (mainly health sciences data from the English Speaking Caribbean); IBECS (Biographic Index on Health Sciences from Spain, a potential source of Spanish language publications from the Caribbean); and the evidence databases of the Pan-American Health Organization and the World Health Organization. The detailed search strategy for Pubmed is given in S1 Appendix. This was adapted as appropriate for the other databases.

The citations identified by the search were downloaded into Endnote (version X4) and the titles and abstracts independently reviewed by two individuals (AN and NU) for potential relevance. Citations were only excluded when both reviewers agreed that they were not relevant. Where doubt existed the citation was retained.

### Data collection, summary measures, and assessment of risk of bias

The full text of all retained articles was sought. Data from articles were abstracted independently by two reviewers in two stages into a Microsoft Access database. The first stage determined whether or not the study met the inclusion criteria, and in the next stage relevant data were abstracted. Differences between first and second data abstractions were resolved at meetings of all the authors.

The data abstraction form was designed to extract key study characteristics and findings relevant to the objective and to enable an assessment of the risk of bias in the study. The content of the data abstraction form was guided by the STROBE statement[12], on reporting observational epidemiology, and by the PRISMA statement on systematic reviews concerning health equity[9]. The reporting of the findings of this systematic review was also guided by the PRISMA statement (S1 Checklist).

All available summary measures by category of each marker of socio-economic position were abstracted from each study, including prevalence, incidence, mean and median, and odds ratios. When available, confidence intervals, p values and measures of dispersion were also collected.

Risk of bias was assessed for each individual relationship. For a relationship to be classified as ‘low risk of bias’ both the study as a whole and the individual relationship had to meet certain pragmatic criteria, as described in the protocol (S1 Appendix). As an example, for a relationship in a cross sectional study to have a low risk of bias, the study itself should be population based and have a response rate of more than 75% with at least 500 participants. In addition, the outcome (e.g. risk factor or diabetes) should be based on objective measures, the relationship assessed controlling for important potential confounders, such as age, and the number of individuals excluded from the analysis must be less than 15%. Three levels of risk of bias were ascribed: low, medium, high/unclear.

### Data Synthesis and Meta-analysis

There was no a-priori plan to undertake meta-analyses, given the anticipated heterogeneity of the studies and settings. In addition, as is shown in the results section, many of the studies were considered to have a high risk of bias. However, on reviewing the findings meta-analyses were considered applicable to summarising the relationships on gender and generalised obesity (I^2^–93%), gender and smoking (I^2^–91%) and gender and diabetes prevalence (I^2^–20%). In these instances there were sufficient studies of low or medium risk of bias to facilitate synthesis. Only population-based cross-sectional studies which measured the outcome as a dichotomous variable were used in the meta-analyses. In the case of diabetes prevalence, only type 2 DM was considered; while for obesity only papers that used an objective measure of body mass index were used. However, smoking was most often measured by self-report. Meta-analysis was undertaken using RevMan 5.2 [13]. RevMan 5.2 was also used to create and examine funnel plots, qualitative examination of which revealed no evidence of publication bias. As no funnel plot included 10 or more studies, formal statistical tests for asymmetry were not applied [14].

## Results

### Study selection

**Fig 1:**
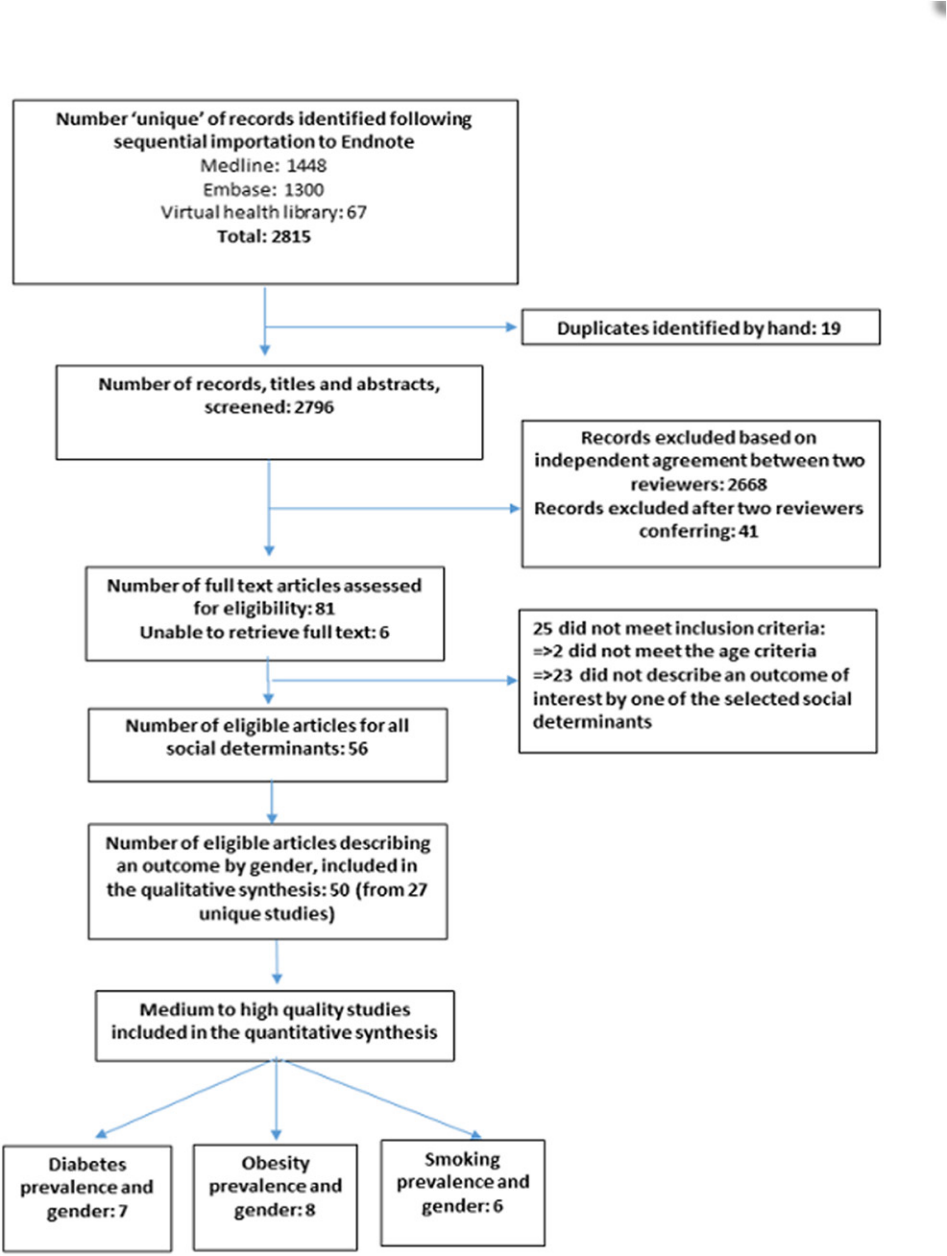
Flowchart of search strategy and article selection.

From our search of seven databases we found 2796 unique records published between the beginning of 2007 and the end of 2013, of which 2709 were excluded after titles and abstracts were screened by two independent assessors (Fig 1) leaving 87 articles for review. Of the 81 articles for which we were able to obtain the full text, a further 25 did not meet the inclusion criteria, leaving 56 articles for review. Three of the remaining six abstracts were based on conference presentations, thus no full text was available and the final unavailable three each originated from an English, Spanish and French speaking territory. For this report the 50 articles which described the distribution of diabetes, risk factors or diabetes control or complications by gender were used in the qualitative synthesis of our results. In addition to producing a narrative synthesis, we performed meta-analyses examining the relationships between gender and diabetes prevalence (n = 7), gender and obesity (n = 8), and gender and smoking (n = 6). In each meta-analysis, Barbosa et al contributed two effect measures from the Study of Health and Well-being in the elderly (SABE) conducted in two Caribbean countries.

### Study Characteristics

Within the 50 articles reviewed there were 27 unique studies, and within these studies we found 114 relationships between the distribution of diabetes mellitus prevalence/incidence *or* diabetes control *or* diabetes complications *or* a related diabetes risk factor *and* gender (Table 1). The studies were conducted in the English-speaking (Bahamas[15], Barbados[16–26], Grenada[27], Jamaica[28–38], Trinidad& Tobago[39–41] and the US virgin islands[42]); French-speaking(Guadeloupe[43–48]); Dutch-speaking (Suriname[49]and Saba [50]) and the Spanish-speaking (Cuba[16, 18, 19, 24, 26, 51–56]and Puerto Rico[57–63]) countries and territories across the Caribbean. As shown in Table 1 the relationships examined provided information either on the distribution of diabetes prevalence/incidence and gender(n = 19) and/or a diabetes-related risk factors by gender: generalized obesity (n = 29), abdominal obesity (n = 19), smoking (n = 15), physical activity (n = 15), aspect of diet (n = 6) and metabolic syndrome (n = 3).There was a scarcity of information(only 3 studies) examining a measure of diabetes control and gender and only one study examining the distribution of a complication of diabetes(mortality). Forty-four of the 50 articles were based on studies with a cross-sectional study design while the remainder utilized a cohort design. Some studies contributed more than one article for example the SABE study—9 articles, Jamaica Health and Lifestyle Survey 2007–2008–4 articles, Barbados Eye Study – 3 articles and Jamaica Survey of Living Conditions—2 articles.

**Table 1:** Characteristics of studies from the Caribbean region describing distribution of selected social determinants by diabetes prevalence, control, complications and diabetes-related risk factors.

Article Title	Study Type/Title	Sample Size	Age ranges	Study-base	Country	Relationships studied (RISK OF BIAS)
Ageyamang, 2009	Cross-sectional	855	12–17	School-based	Suriname	Obesity (BMI) and gender (HIGH); Physical activity and gender (HIGH)
Anderson, 2011	Cross-sectional	857	24–74	Population-based	Jamaica	Diabetes prevalence and gender (HIGH; Impaired fasting glucose and gender (HIGH); Obesity (BMI) and gender (MEDIUM); Obesity (WC) and gender MEDIUM); Smoking and gender (HIGH); Aspect of diet and gender (HIGH)
Andrade, 2009	Cross-sectional/ Study of Health and Well-being in the Elderly in Latin America and the Caribbean(SABE)	13753	60+	Population-based	Barbados & Cuba	Diabetes prevalence and gender (HIGH)
Apparico, 2007	Cross-sectional	132	27–81	Health facility	Trinidad	Diabetes control (HbA1c) by gender(HIGH)
Atallah, 2007	Cross-sectional/ Prevalence of Hypertension in a Population Précaire Guadeloupéenne	2420	18–69	Health facility	Guadeloupe	Obesity (BMI) and gender (HIGH); Physical activity and gender (HIGH); Smoking and gender (HIGH)
Atallah, 2011	Cross-sectional/ APHYGUAD study	685	18–74	Population-based	Guadelope	Physical activity and gender(HIGH)
Barbosa, 2010	Cross-sectional/ SABE	1508	60+	Population-based	Barbados	Diabetes prevalence and gender(MEDIUM); Obesity (BMI) and gender (LOW); Obesity (WC) and gender (LOW); Physical activity and gender (HIGH); Smoking and gender (MEDIUM)
Barbosa, 2011	Cross-sectional/ SABE	3413	60+	Population-based	Cuba & Barbados	Diabetes prevalence and gender(MEDIUM); Obesity (BMI) and gender (LOW); Obesity (WC) and gender (LOW); Physical activity and gender (HIGH); Smoking and gender (MEDIUM)
Barcelo, 2007	Cross-sectional/ SABE	13753	60+	Population-based	Barbados & Cuba	Diabetes prevalence and gender (MEDIUM); Obesity (BMI) and gender(LOW); Obesity (WC) and gender (LOW); Smoking and gender (MEDIUM)
Barcelo, 2007	Cohort	504	>0	Population-based	Cuba	Mortality and gender (HIGH)
Barrett, 2013	Cross-sectional	276	14–19	School-based	Jamaica	Obesity (BMI) and gender (HIGH); Obesity (WC) and gender (HIGH)
Block, 2012	Cross-sectional/Grenada Heart Project, 2005–2007	2017	18–104	Population-based	Grenada (Petit Martinique & Carriacou)	Diabetes prevalence and gender (MEDIUM); Physical activity and gender (HIGH); Obesity (BMI) and gender (MEDIUM); Smoking and gender (MEDIUM); Obesity (WC) and gender (MEDIUM); Metabolic syndrome and gender(HIGH); Aspect of diet and gender(HIGH)
Bourne, 2011	Cross-sectional/ Jamaica Surveys of Living Conditions(JSLC)	31,801	>18	Population-based	Jamaica	Diabetes prevalence and gender(HIGH)
Boyne, 2010	Cross-sectional/ International Collaborative Study of Hypertension in Blacks (ICSHIB)	393	25–74	Population-based	Jamaica	Obesity (BMI) and gender(HIGH); Obesity (WC) and gender (HIGH)
Brathwaite, N, 2011	Cross-sectional	2469	21–60	Population-based	Bahamas	Obesity (BMI) and gender (HIGH)
Chadee, 2013	Cross-sectional	15649	>17	Institution-based	Trinidad and Tobago	Diabetes prevalence and gender (HIGH)
Colon-Lopez, 2013	Cross-sectional/Puerto Rico Health Information National Trends Survey (HINTS-PR)	593	>18	Population-based	Puerto Rico	Aspect of diet and gender (LOW)
Cruz, 2013	Cross-sectional	275	≥ 21	Tertiary Institution-based	Puerto Rico	Physical activity and gender (HIGH)
Cumberbatch, 2011	Cross-sectional/ Jamaica Health and Lifestyle Survey 2007–2008 (JHLS 2007–8)	2,432	15–74	Population-based	Jamaica	Diabetes prevalence and gender (HIGH); Physical activity and gender (HIGH); Obesity (BMI) and gender (HIGH); Smoking and gender (HIGH)
Cunningham-Myrie, 2013	Cross-sectional/JHLS 2007–8)	2848	15–74	Population-based	Jamaica	Obesity (BMI) and gender (MEDIUM); Obesity (WC) and gender (MEDIUM); Physical activity and gender (HIGH); Diabetes prevalence and gender (MEDIUM)
da Silva Coquiera, 2009	Cross-sectional/SABE	1905	>60	Population-based	Cuba	Obesity (BMI) and gender (LOW)
da Silva Coqueiro, R., 2010	Cross-sectional/SABE	1905	≥ 60	Population-based	Cuba	Obesity (BMI) and gender(MEDIUM)
Diaz Sanchez, 2009	Cross-sectional/ Second National Survey on Risk Factors and Chronic Diseases (ENFRENT II)	19519	≥ 20	Population-based	Cuba	Obesity (BMI) and gender (MEDIUM); Obesity (WC) and gender (MEDIUM)
Fabelo, 2013	Cross-sectional	108	(No range given)	Institution-based	Cuba	Smoking and gender (HIGH)
Fabian, 2013	Cross-sectional	275	≥ 21	Tertiary Institution-based	Puerto Rico	Aspect of diet and gender (HIGH)
Ferguson, 2008	Cross-sectional/ Jamaica Healthy Lifestyle Survey 2000–2001 (JHLS 2000–2001)	2012	15–74	Population-based	Jamaica	Diabetes prevalence and gender (HIGH/UNCLEAR); Obesity (BMI) and gender (HIGH/UNCLEAR); Obesity (WC) and gender (HIGH/UNCLEAR); Physical activity and gender (HIGH); Smoking and gender (HIGH)
Ferguson, 2010a	Prospective Cohort /Spanish Town	708	25–74	Population-based	Jamaica	Diabetes prevalence and gender(HIGH); Obesity (BMI) and gender (HIGH); Obesity (WC) and gender (HIGH); Smoking and gender (HIGH)
Ferguson, 2010b	Cohort study/1986 Jamaica Birth Cohort	839	18–20	Population-based	Jamaica	Obesity (BMI) and gender(MEDIUM); Obesity (WC) and gender (MEDIUM); Physical activity and gender (HIGH); Impaired fasting glucose and gender (MEDIUM); Metabolic syndrome and gender (MEDIUM)
Ferguson, 2011	Cross-sectional/ (JHLS 2007–8)	2848	15–74	Population-based	Jamaica	Diabetes prevalence and gender (HIGH); Obesity (BMI) and gender (HIGH/UNCLEAR); Obesity (WC) and gender (HIGH/UNCLEAR); Physical activity and gender (HIGH); Smoking and gender (HIGH); Diabetes control(FG) and gender (HIGH/UNCLEAR)
Foucan,2007	Cross-sectional	966	18–74	Health facility	Guadelope	Obesity (BMI) and gender (HIGH); Obesity (WC) and gender (HIGH); Impaired fasting glucose and gender(HIGH)
Garza, 2011	Cross-sectional/Asthma Depression and Anxiety in Puerto Rican Youth	872	10–19	Population-based	Puerto Rico	Obesity (BMI) and gender (HIGH);
Gonzalez, 2013	Cross-sectional	274	21–53	Tertiary Institution-based	Puerto Rico	Smoking and gender (HIGH)
Kim, 2007	Cross-sectional/SABE	Cuba-1904, Bdos-1508	>60	Population-based	Barbados Cuba	Smoking and gender(HIGH)
Laborde, 2013	Cross-sectional/Behavioral Risk Factor Surveillance System (2006,2010)	6025	>18	Population-based	Puerto Rico	Obesity (BMI) and gender (MEDIUM)
Latchan, 2010	Cross-sectional	688	18–92	Health Facility	Trinidad	Obesity (WC) and gender (HIGH); Diabetes prevalence and gender (HIGH)
Llibre, 2011	Prospective cohort/10/66 Study	3015	65+	Population-based	Cuba	Diabetes prevalence and gender (MEDIUM)
Modeste, 2007	Cross-sectional	407	18–74	Faith-based institution	Barbados	Physical activity and gender (HIGH); Diabetes prevalence and gender (HIGH)
Nam, 2012	Cross-sectional/ SABE	Bdos 994 Cuba 1073	60+	Population-based	Barbados and Cuba	Diabetes prevalence and gender (MEDIUM); Obesity (BMI) and gender (MEDIUM); Obesity (WC) and gender (MEDIUM); Smoking and gender (MEDIUM)
Naranjo, 2013	Cross-sectional	620	>18	Health facility-based	Cuba	Obesity(WC) and gender (HIGH)
Nemesure, 2007	Cohort study/ Barbados Eye Study (BES)	4314	40 and older	Population-based	Barbados	Obesity (BMI) and gender (LOW)
Nemesure 2008a	Cohort study/ (BES)	2793	40–84	Population-based	Barbados	Obesity incidence (BMI) and gender (MEDIUM); Diabetes incidence and gender (MEDIUM)
Nemesure, 2008b	Cohort study/ (BES)	1790	40–84	Population-based	Barbados	Obesity incidence (BMI) and gender (MEDIUM)
Nunez, 2011	Cross-sectional	53	26–80	Health facility based	US Virgin Islands	Diabetes control (HbA1c) and gender (HIGH)
Palloni, 2007	Cross-sectional/ SABE	3713	60+	Population-based	Barbados & Cuba	Diabetes prevalence and gender (HIGH)
Perez, 2008	Cross-sectional	867	21–79	Population-based	Puerto Rico	Impaired fasting glucose and gender (LOW); Obesity (WC) and gender (LOW); Metabolic syndrome and gender (MEDIUM
Sinnapah, 2009	Cross-sectional	122	17–66	Health facility based	Guadelope	Physical activity and gender (HIGH); Obesity (BMI) and gender (HIGH); Aspect of diet and gender (HIGH)
Sinnapah, 2009	Cross-sectional	780	10–18	School-based	Guadelope	Physical activity and gender (HIGH); Obesity (BMI) and gender (HIGH)
Sinnapah, 2009	Cross-sectional	720	11–17	School-based	Guadelope	Obesity (BMI) and gender (HIGH); Obesity (WC) and gender (HIGH)
Soloway, 2009	Cross-sectional	278	18–91	Population-based/Convenience sample	Saba(Netherlands)	Obesity (BMI) and gender (HIGH)
Tulloch-Reid, 2013	Cross-sectional/JHLS 2007–8	1432	40–74	Population-based	Jamaica	Smoking and gender (HIGH)

### Risk of Bias within Studies

Over half of the relationships studied (74) were found to have a high risk of bias (Table 1). In some cases, the high risk of bias was due to the lack of a population based study design, with this being the case in 17 of the 50 articles identified. In these 17 articles the studies were based in health care facilities (n = 7), schools (n = 7), workplaces (n = 2) and a church (n = 1). We also considered cross-sectional studies with fewer than 500 persons and/or low (<50%)) or unclear response rates to have high risk of bias (n = 11).

There were several articles where the study as a whole was judged to have a low or medium risk of bias but where the relationship with gender was not adjusted for age. In some studies the health outcomes and risk factors were measured using subjective measures such as self-report where an objective measure is the accepted standard. Of the 19 articles that reported gender and diabetes prevalence, 9 used self-reported diagnosis only i.e. did not measure glucose. For physical activity assessment all studies used a questionnaire. The questionnaires included the International Physical Activity Questionnaire (IPAQ), 24 hour recall of 3 consecutive days, PAHO STEPS survey and single questions such as “How many times have you been active for 3 or more times per week in the past 12 months?” Smoking status was also exclusively assessed by self-report. With respect to obesity, it was based on measured BMI in 28 articles, and in two articles self-reported height and weight were used to calculate BMI.

### Results of Individuals Studies

#### Gender and Diabetes prevalence

**Fig 2:**
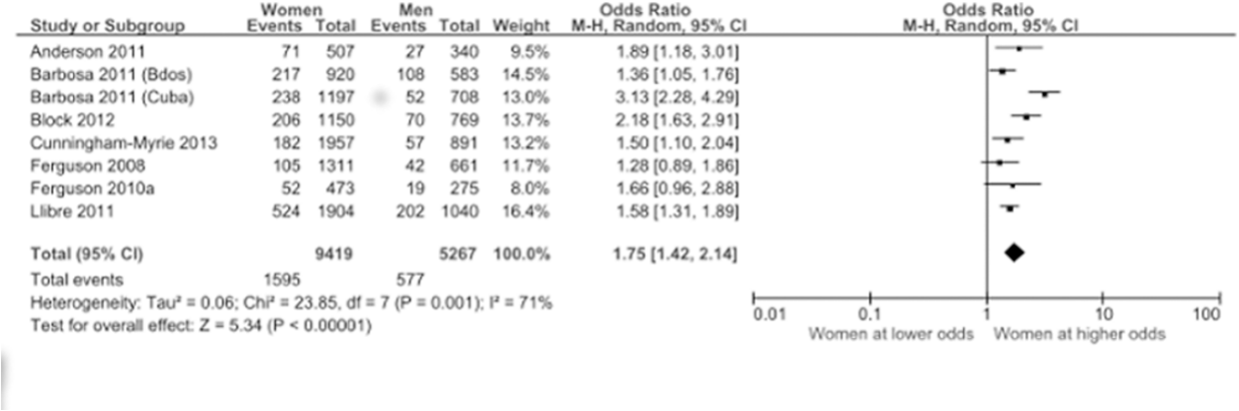
Relationship between gender and diabetes prevalence. Articles assessing diabetes prevalence by objective measures and self-report were included.

**Fig 3:**
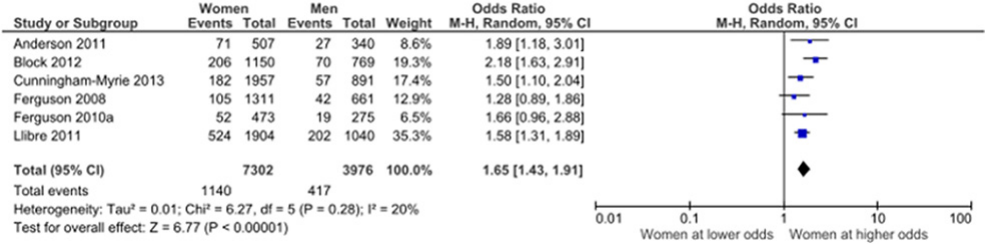
Gender and objective diabetes prevalence. Only articles assessing diabetes prevalence by objective measures were included.

There were 19 articles from 12 unique studies which examined the relationship between diabetes prevalence and gender, 9 of these were based on population-based studies (Table 2).All 9 studies found a higher prevalence of DM in women than in men although in 2 studies this was not statistically significant (p>0.05), while in another 3 there was no assessment of type 1 error. In the meta-analysis, we included population-based studies that used objective and self-reported measures of diabetes prevalence and were considered low to medium risk of bias. The summary result of these studies indicates that women were more likely to have diabetes than men (OR 1.75, 95% CI 1.42, 2.14)(Fig 2), with subgroup analysis including only those population-based studies which used objective measures of diabetes mellitus finding a similar, if slightly weaker, relationship(OR 1.65, 95% CI 1.43, 1.91) (Fig 3). Four of these studies were from Jamaica, while the largest was from Cuba where women were 1.58 times more likely to have diabetes than men. (OR 1.58, 95% CI 1.31, 1.89).There was significant heterogeneity in combining the studies with I^2^ values of 71% and 20% for the combined and subgroup analysis respectively.

**Table 2:** Characteristics of studies providing information on the distribution of diabetes prevalence by gender.

Article Title /(Study name)	Sample Size	Age ranges	Country	Risk of bias assessment
Anderson 2011	857	24–74	Jamaica	Cross-sectional; population-based; Response rate: 62%; 2006 WHO criteria were used to classify subjects according to their glucose tolerance status: diabetes = fasting plasma glucose ≥ 7 0 mmol/l or 2 h postprandial glucose ≥ 11 1 mmol/l).
Block 2012	2017	18–104	Grenada (Petit Martinique & Carriacou)	Cross-sectional study; Population-based; 64% response rate; A diagnosis of diabetes was based on participant self-report or fasting glucose of 140 mg/dL.
Cumberbatch 2011 (JHLS 2007–8)^a^	2432	15–74	Jamaica	Cross-sectional study; Population-based; 98% response rate; Diabetes measured by fasting capillary glucose ≥6.1 mmol/L or if they responded yes to the question, “Have you been prescribed medication for your diabetes?”
Cunnngham-Myrie, 2013 (JHLS 2007–8)^a^	2848	15–74	Jamaica	Cross-sectional study; Population-based; 98% response rate; Diabetes measured by fasting capillary glucose ≥6.1 mmol/L or if they responded yes to the question, “Have you been prescribed medication for your diabetes?”
Ferguson 2008 (JHLS 2000–2001)^b^	2012	15–74	Jamaica	Cross-sectional survey; Population-based; 87.6% response rate; Diabetes mellitus was defined as having a fasting glucose of greater than or equal to 6.1 mmol/L or being on treatment for diabetes.
Ferguson 2010a	708	25–74	Jamaica	Cohort study; 54% follow-up of original cohort; Data analysed for only 45% of eligible participants at baseline; Diabetes mellitus was defined according to theAmerican Diabetes Association 1997 criteria as fasting glucose ≥ 7.0 mmol/L or two-hour post challenge glucose of ≥ 11.1 mmol/L or taking medication for diabetes mellitus.
Llibre 2011	3015	65+	Cuba	Cross-sectional; Population-based; Response rate 97.6%; Diabetes mellitus, diagnosed in two ways: 1) self-report of physician diagnosis of diabetes (“Have you ever been told you have diabetes? Did you start treatment? Are you still being treated?”); and/or 2) fasting glucose of _7.0 mmol/L confirmed on two different days
Ferguson 2011 (JHLS 2007–8)^a^	2848	15–74	Jamaica	Cross-sectional study; Population-based; 98% response rate; Diabetes measured by fasting capillary glucose ≥6.1 mmol/L or if they responded yes to the question, “Haveyou been prescribed medication for your diabetes?”
*Andrade 2009 (SABE)^c^	Bdos 1508 Cuba 1903	60+	Barbados & Cuba	Cross-sectional; Population-based; 81% response rate in Barbados, 95.3% in Cuba.Diabetes measured by self-report, No adjustment for confounders
*Barbosa 2010 (SABE)^c^	1508	60+	Barbados	Cross-sectional, Population-based; Response rate 81%; DM as measured by self-report via a questionnaire.
*Barbosa 2011 (SABE)^c^	Bdos1508 Cuba 1905	60+	Cuba & Barbados	Cross-sectional; Population-based; Diabetes measured by self-report, 81% response rate in Barbados, 95.3% in Cuba
*Barcelo 2007 (SABE)^c^	Bdos 1508 Cuba 1903	60+	Barbados & Cuba	Cross-sectional; Population-based; 81% response rate in Barbados, 95.3% in Cuba.Diabetes measured by self-report, No adjustment for confounders
*Nam 2012 (SABE)^c^	Bdos 994 Cuba 1073	65+	Barbados and Cuba	Cross-sectional; Diabetes measured by self-report, 81% response rate in Barbados, 95.3% in Cuba no adj for confounders
*Palloni 2007 (SABE)^c^	Bdos 1508 Cuba 1903	60+	Barbados & Cuba	Cross-sectional; Diabetes measured by self-report, population-based, 81% response rate in Barbados, 95.3% in Cuba no adjustment for confounders
*Bourne 2011 (JSLC)^d^	31,801	No ages given	Jamaica	Cross-sectional study; Population-based; JSLC for 2007 had a response rate 73.8%, and for 2002 it was 70.3%; diabetes measured by self-report via questionnaire; Inconsistent assessment of point estimate;
*Bourne, 2010 (JSLC) ^d^	15,260(2002) 3,322(2007)	>18	Jamaica	Cross-sectional study; Population-based; JSLC for 2007 had a response rate 73.8%, and for 2002 it was 70.3%; diabetes measured by self-report via questionnaire; Inconsistent assessment of point estimate;
**Latchan, 2010	688	18–92	Trinidad	Cross-sectional; Health Facility; Response rate not given; patient was classified as having diabetes if two of the following criteria were met: (1) a fasting blood sugar ≥ 7mmol/L or a 2-hour post-prandial reading of ≥ 11.1mmol/L following a 75 g glucose load, and (2) currently receiving any combination of lifestyle interventions, oral antidiabetic drugs orinsulin therapy.
**Modeste, 2007	407	18–74	Barbados	Cross-sectional; Faith-based institution; Response rate = 66.9%; DM measured as Fasting venous plasma glucose level of 7.8 mmol/L (140 mg/dL) or higher was used to classify a person as diabetic, or if a person self-reported a physician’s diagnosis of diabetes.
**Chadee, 2013	15649	>17 years	Trinidad and Tobago	Cross-sectional; Database of State-managed financial assistance program; Usable data for 68%; Diabetes measured by self-report;

^a^ Jamaica Health and Lifestyle Survey 2007–8.

^b^ Jamaica Health and lifestyle Survey 2000–1.

^c^ Study of Health and Well-being in the Elderly in Latin America and the Caribbean.

^d^ Jamaica Survey of Living Conditions.

*These studies use self-report to diagnose diabetes.

**These studies are not population-based.

#### Gender and risk factors

**Fig 4:**
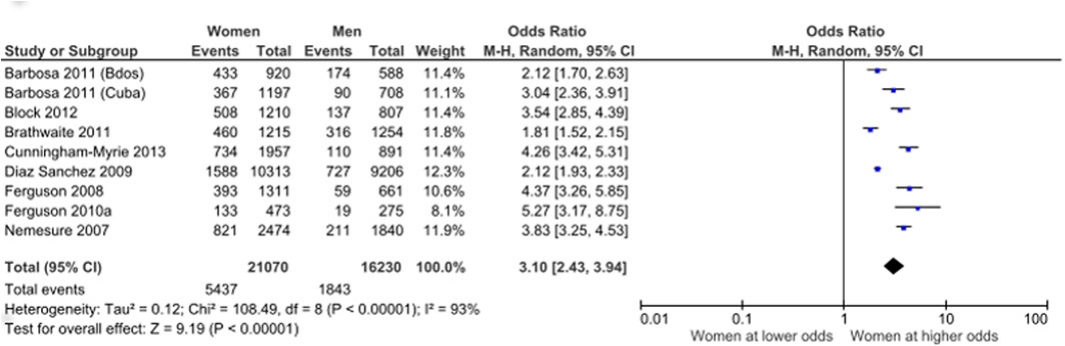
Relationship between gender and obesity. Only articles assessing obesity using an objective measure of body mass index (BMI) were included.

We examined the relationships between gender and various diabetes risk factors. There were 30 papers describing the relationship between gender and obesity as assessed by body mass index (BMI) from Jamaica (n = 7), Barbados (n = 6), Cuba (n = 6), Guadeloupe (n = 5), Suriname (n = 2), Puerto Rico (n = 2) Bahamas, Grenada, Saba and Trinidad (1 each)—3 papers covered both Cuba and Barbados (S2 Table). Studies that were population-based, measured body mass index by an objective standard protocol, adjusted for age or considered subjects in a narrow age range were assessed as having a low risk of bias. Men tended to have lower mean BMI values than women and this was true of all studies reporting this statistic in the adult population. Block et al [27] demonstrated that women in Grenada had higher mean BMI than men in each age group. Studies from Guadelope [47, 48]and Suriname[49] examined mean BMI in adolescent populations and found similar mean BMI values in girls and boys. Of the 16 articles from 13 unique studies which provided prevalence rates for obesity, 8 were population-based and measured obesity objectively (Table 3). These eight studies all found higher rates of obesity in women compared to men and when meta-analysed, provided an odds ratio in favour of women of 3.10 (95% CI 2.43, 3.94)(Fig 4). There were 18 articles that examined gender and abdominal obesity as measured using waist circumference and/or waist-to-hip ratio. Eight of these articles were assessed as having high risk of bias, while the remaining ten consistently reported higher rates of abdominal obesity in women compared to men.

**Table 3:** Characteristics of studies providing information on the distribution of obesity prevalence by gender.

Article Title	Sample Size	Country	Age ranges	Prevalence Males (%)	Prevalence Females (%)	Risk of bias assessment
Atallah, 2007	2420	Guadelope	18–69	12.3	29.0	Cross-sectional; Health-facility-based; Obesity measured objectively. Defined as BMI>30
Barbosa 2010	1508	Barbados	60–74	24.7	47.2	Cross-sectional; Population-based; 81% response rate in Barbados, 95.3% in Cuba. BMI objectively measured. BMI measured as >28kg/m2
Barbosa 2011	1508 / 1905	Barbados / Cuba	Over 60	Barbados: 29.6 / Cuba: 12.7	Barbados: 47.2 / Cuba: 30.7	Cross-sectional; Population-based; 81% response rate in Barbados, 95.3% in Cuba; BMI objectively measured.
Barrett 2013	276	Jamaica	14–19	20	24	Cross-sectional; School-based; Response rate 92%; BMI objectively measured; Adjustments for confounding made.
Block, 2012	2017	Grenada	18–104	17.0	42.0	Cross-sectional; Population-based; 64% response rate; BMI objectively measured
Brathwaite, 2011	2469	Bahamas	21 to 60	25.2	37.9	Cross-sectional; population-based; Coverage rate of 64%; BMI objectively measured;
Cunningham-Myrie, 2013	2848	Jamaica	15–74	12.3 (9.4, 15.9)	37.5 (34.6, 40.5)	Cross-sectional; Population-based; Response rate 98%, BMI objectively measured
da Silva Coquiero, 2010	1905	Cuba	> = 60	12.7	30.7	Cross-sectional; Population-based; Response rate 95.3%, BMI objectively measured.
Diaz Sanchez, 2009	19519	Cuba	> = 20	7.9; (95% CI: 7.3–8.6).	15.4; (95% CI: 14.5–16.3)	Cross-sectional; population-based; Response rate not stated; BMI objectively measured;
Ferguson, 2008	2045	Jamaica	15–74	9.0 (95% CI) (6.8–11.3)	30.0 95% CI (26.8–33.2)	Cross-sectional; population-based; crude presentation of RF; Response rate 87.6%; BMI objectively measured.
Ferguson, 2010a	708	Jamaica	25–74	6.9	28.2	Population based; objective measures, (and no missing data for this relationship); lack of adjustment for confounders
Ferguson, 2011	2848	Jamaica	15–74	12.4	37.7	Cross-sectional; population based; Not age adjusted and missing data unclear (data weighted for missing values); BMI objectively measured.
Foucan, 2007	966	Guadelope	18–74	11.2	14.8	Cross-sectional, health-facility, No missing values, objective measures taken, no response rate reported, no age adjustment
Garza, 2011	436	Puerto Rico	10–19	45.0	33.8	Cross-sectional; population-based; Response rate 79.5%; BMI measured via self-report of height and weight
Laborde, 2013	6025	Puerto Rico	>18	27.3	32.2	Cross-sectional; population-based; Response rate 61%; BMI measured by self-report of height and weight
Nemesure, 2007	4314	Barbados	40–84	11.5	33.2	Cohort study(baseline data used here); Population-based; 84% response rate. BMI objectively measured.

Fifteen articles described the relationship between physical activity and gender(S1 Table), all of which were assessed as having a high risk of bias as samples were either not population-based (6) or obtained by convenience sampling(1) or measured physical activity by self-report via a questionnaire (15). All studies except one[46]reported higher levels of physical activity among men and/or higher levels of sedentary time among women.

**Fig 5:**
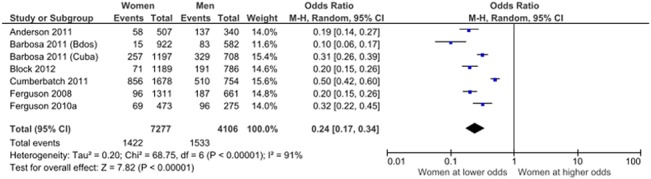
Relationship between gender and smoking. Data from population-based cross-sectional studies.

Men were more likely to smoke than women. There were 15 articles drawn from 9 studies which examined the relationship between gender and smoking. Six of these studies were population-based and were used in a meta-analysis using a random effects model to summarize the relationship between gender and smoking (Table 4). There was significant heterogeneity (p<0.001) between studies (I^2^ = 91%), and a random effects model was used. The odds of smoking among women was 0.24 times that of smoking among men, 95% CI (0.17, 0.34) (Fig 5).

**Table 4:** Characteristics of population-based cross-sectional and cohort studies providing information on the distribution of smoking prevalence by gender.

Author, year published	Sample size	Country	Sex (% Male)	Age range	Risk of bias assessment
Anderson, 2011	857	Jamaica	40.1%	24–74	Response rate 62%; No adjustment for confounders
Block, 2012	2017	Grenada	39.8%	18–104	Response rate 64%; Adjustment for age; No assessment of precision of estimates
Cumberbatch, 2011	2848	Jamaica	31.0%	15–74	Response rate 98%; No adjustment for confounders
Ferguson, 2008	2012	Jamaica	33.5%	15–74	Response rate 87.6%; No adjustment for confounders
Ferguson, 2010a	708	Jamaica	36.8%	25–74	54% follow-up of original cohort; No adjustment for confounders
Kim, 2008	1504 1904	Barbados Cuba	38.7% 37.2%	> = 60	Response rate 81% in Barbados, 95.3% in Cuba; Adjusted for age

There were five articles in which one or more aspects of diet were assessed and described by gender. The most robust of these studies was conducted in Puerto Rico where researchers found a slightly higher percentage of men consumed at least 5 servings of fruit and vegetables per day than women (8.2% vs. 6.3%) but this difference was not significant (p = 0.23). In a population based study from Jamaica, Anderson[34] found that men had a higher total energy intake than women and similar percentage energy intakes from carbohydrates, while women 32·8% (95% CI 32·1, 33·4) had slightly higher energy percentages from fat than men (31·8% 95% CI 31·0, 32·7), and men had significantly higher percentages from protein. Block et al[27], who also conducted a population-based study, found that younger women in Grenada were less likely to eat 2 or more servings of fish per week than younger men but this gender difference was not seen in older ages. Men and women had similar patterns of fruit and vegetable consumption. A small health facility-based study conducted in Guadeloupe[46] found that men and women had similar percentage energy intakes from protein, lipid and carbohydrates. A university-based study in Puerto Rico [58]evaluated the adequacy of dietary elements that must be supplied in sufficient amounts to guarantee a ‘healthy diet’. They found very little difference between men and women in the aspects of diet they assessed.

We found 3 population-based cross-sectional studies that examined the relationship between gender and metabolic syndrome. Two of these from Grenada and Jamaica [27, 36]showed that women had higher rates of metabolic syndrome while the third was done in Puerto Rico and found men 42.1% (36.5–47.7), to have slightly higher rates than women 36.4% (32.7–40.2). For only one of these studies (Grenada)[27] was the difference statistically significant (women had a metabolic syndrome prevalence of 36% versus 17% for men (P <. 001)). The Jamaican study defined the metabolic syndrome using the International Consensus Criteria [64]while in both Grenada and Puerto Rico they used the NCEP-ATP III criteria[65].

#### Gender and Diabetes control and complications

There was a paucity of studies describing the distribution of diabetes control or complications by gender(S3 Table). We found three articles [31, 39, 42] in which the outcome was diabetes control and all of which were classified as having a high risk of bias. Two of these studies used HbA1c while the third used fasting glucose as a measure of control. They each showed that women were more likely to have poor control or be uncontrolled compared to men. Only in one was statistical significance attained where men had significantly tighter glycaemic control than women(p = 0.0271)[42]. Only one study described the relationship between mortality and gender and found similar mortality rates for the two sexes for persons with type 1 diabetes[51].

## Discussion

### Summary of the evidence

We undertook a systematic review of the distribution by gender of diabetes, risk factors for type 2 diabetes, and diabetes control and complications in the Caribbean, based on studies published between 2007 and the end of 2013. The choice of 2007 as a start point was based on a major political initiative within the Caribbean to take action on chronic diseases[66], and this work is in part designed to help inform policy and research in support of this initiative.

We found a statistically significant association between gender and diabetes in the Caribbean region, with women being at more than one and a half times greater risk than men. The prevalence and incidence data we examined did not distinguish between type 1 and type 2 diabetes, but as type 2 diabetes represents 90 to 95% of all diabetes[7, 67] it is reasonable to conclude that the gender difference is largely based on differences in type 2 diabetes.

Obesity is by far the strongest modifiable risk factor for type 2 diabetes[68], and this was three times higher in women. In addition, the data we found overwhelmingly suggested they were less physically active. Of the risk factors for type 2 diabetes that we found data on, the only one that was more common in men was tobacco smoking, where men were at four times greater risk. We found only three studies describing glycemic control by gender. In each of these studies a high risk of bias was found, thus we regarded the better control found in men as inconclusive., Overall, our findings suggest greater risk of diabetes, greater prevalence of diabetes, and potentially poorer diabetes control in women compared to men.

The relationships we found between gender and diabetes prevalence, and between gender and obesity, were consistent across different settings in the Caribbean, older and younger adults, and based on studies with a low to medium risk of bias. The relationships were found in population based studies, with good response rates, and with objective measures e.g. fasting glucose, and measured height and weight. Our finding that women are at significantly greater risk of diabetes is in marked contrast to what is described in most populations, where the prevalence is either similar between the sexes or higher in men. Indeed evidence suggests that for a given level of obesity adult men are at greater risk of type 2 diabetes than women[8]. While the reasons for this are not fully understood they are likely to include a greater tendency to abdominal and hepatic fat deposition in men. This is likely to be part of the explanation as to why in many populations the prevalence of diabetes is similar between men and women despite higher levels of obesity in women[8]. Another factor that may increase a tendency in many populations to type 2 diabetes in men compared to women is a much higher prevalence of tobacco smoking in men[69]. Tobacco smoking is associated with a 40 to 50% increase in the risk of type 2 diabetes[70]. While smoking in the Caribbean is on average much higher in men than women, its prevalence in men is typically between 10 and 20%, lower than most world regions [69].

The only other world region in which an excess of diabetes in adult women compared to men has been described, as far as we are aware, is Southern Africa. A systematic review and meta-analysis of diabetes prevalence studies in sub-Saharan Africa found a male excess in diabetes prevalence in Central and Eastern Africa but a female excess in Southern Africa (OR 1.25, 95% CIs 1.09, 1.45)[71]. Estimates of the prevalence of obesity from the Global Burden of Disease study[72] suggest a modest excess (1.5% to 4.4%) of obesity in females compared to males in Central and Eastern Africa, but a much larger excess 26% (11.7% men vs 37.7% women), in Southern Africa, which is similar to what we have found in the Caribbean. We postulate therefore that this degree of excess is in part responsible for the higher prevalence of type 2 diabetes in women compared to men which we found.

Finally, it is relevant to note that there is evidence that the strength of the relationship between increased BMI and the risk of type 2 diabetes differs between men and women. Thus, while the incidence (absolute risk) of type 2 diabetes tends to be higher in men than in women at all BMI levels, the relative risk of a raised BMI compared to a normal BMI tends to be greater in women than in men. For example, a systematic review and meta-analysis describes a pooled relative risk of 6.4 (95% CIs 5.6–8.2) in men for BMI of 30 and above compared to less than 25, and pooled relative risk of 12.4 (9.0–12.1) in women [73]. This seems partly to reflect the fact that at normal BMI levels (e.g. < 25) the incidence of diabetes is substantially lower in women than in men[74].

While overweight/obesity is the strongest single risk factor for type 2 diabetes, other factors may also play an important role in the female excess we have found. In particular, physical inactivity, independent of its role in obesity[75], is a risk factor for type 2 diabetes, and we found evidence (albeit not suitable for meta-analysis) of higher levels of physical inactivity in women compared to men across several settings in the Caribbean.

Our findings have two broad implications. The first is around understanding the gender distribution of type 2 diabetes within populations. We suggest that our findings provide some insight into the relative distribution of risk factors between genders, particular obesity, needed to produce a population level female excess of type 2 diabetes. In both the Caribbean and Southern Africa women are about three times more likely to be obese, with an absolute difference of 20 to 40% in prevalence. It may be speculated that the gender disparity in both regions is a manifestation of a shared cultural heritage as well as dietary and lifestyle practices. There has been a long held tradition in the Caribbean that obesity is healthy and that larger, more full bodied, women are preferred[76]. Some have speculated that this may be related to the former custom amongst some groups in Africa of hosting brides-to-be in fattening huts to please their future partner[76]. Studies conducted in Barbados on obesity attitudes and preferences indicated a change in male preference towards a normal or near-normal female figure but a continuing increase in prevalence and acceptance by females of obesity [77]. It is conceivable that with economic development other regions of Africa, and indeed other developing regions outside Africa, may also see a marked female obesity excess and that in these regions type 2 diabetes will be significantly more common in women than men.

The other broad implication is around research and policy for the prevention of type 2 diabetes. In particular, research is needed to better understand why in some populations, such as in the Caribbean, women are at so much greater risk of obesity, and what can be done to reduce that risk. The mechanisms when elucidated are bound to be complex and may revolve around women’s roles and responsibilities in society. Health promotion campaigns that are based on the research undertaken and mechanisms elucidated can then be appropriately conceptualized. Similar considerations apply to physical inactivity. Policy makers may need to consider measures that specifically target the physical activity needs of women, and further research is required to understand properly how different these needs are relative to those of men. Our findings suggest that the determinants of obesity and physical inactivity differ substantially between men and women in the Caribbean and that preventive policies must separately target and monitor their impact on men and women.

### Limitations

The relationships we abstracted on diabetes prevalence, obesity, physical inactivity and smoking by gender are consistent in direction across studies and settings. Nonetheless the number of studies of low to medium risk of bias we identified was small. In addition, none of the studies were concerned specifically with examining the distribution of their findings by gender thus we are unable to comment on whether this finding of diabetes excess in women differed by ethnic group, education occupation or income within the Caribbean. For example, none of the studies we found adjusted for differences in risk factors or other social determinants between men and women in describing diabetes prevalence, or investigated potential underlying determinants for gender differences in obesity.

While our search strategy was broad, and included through the Virtual Health Library databases that record some ‘grey literature’, such as government reports and PhD theses, we did not have the resources to undertake more extensive grey literature searching.

### Conclusions

We have described in the Caribbean region a 50% excess risk of diabetes in women, associated with an approximate threefold excess risk of obesity, and consistently lower levels of physical activity. As far as we are aware no other world region has such an extreme female excess of diabetes, with the only other region showing a similar pattern being Southern Africa. It is reasonable to speculate that with economic development other developing world regions, such as other parts of Africa, may see similar differences between men and women. Our findings have major implications for research to understand the reasons for gendered differences in the risk of diabetes, and for policy aimed at its prevention.

## Supporting Information

S1 Checklist PRISMA Checklist. (PDF)

S1 Appendix Systematic review protocol. (DOCX)

S1 Table Studies examining gender and physical activity. Only cross-sectional studies have been included.
(DOCX)

S2 Table Studies examining obesity by gender. Mean body mass index has been used as the measure of obesity.
(DOCX)

S3 Table Studies describing diabetes control and gender. (DOCX)
