# Supplementary material for: Female Gender Is a Social Determinant of Diabetes in the Caribbean: A Systematic Review and Meta-Analysis
Source: PLoS One. 2015 May 21;10(5):e0126799. doi: 10.1371/journal.pone.0126799 (PMC4440736; doi:10.1371/journal.pone.0126799)
Supplement: S1 Appendix — (DOCX) [file pone.0126799.s002.docx]

**Title:** Health inequities of diabetes, its risk factors, and adverse outcomes in populations living in the Caribbean: a systematic review

**Rationale**

The United Nations High Level Meeting in 2011 on non-communicable diseases acknowledged that the effective prevention of diabetes and related chronic diseases will require population wide approaches that address their underlying, social, determinants (1). Related to this is the ethical and public health challenge of identifying and addressing health inequities between populations and groups (2), to which diabetes is known to contribute substantially. To date, there has been no published systematic review of research conducted in the Caribbean that examines the social determinants of diabetes, a major contributor to death and disability in the region (3, 4). Such a review should inform preventive strategies for diabetes and its complications within the region, and identify areas for further research.

**Theoretical framework**

The planning of this review was guided by the analytical framework used to examine the social determinants of specific conditions in the work of the WHO Commission on the Social Determinants of Health (2). The framework has five levels and three dimensions, as shown in the figure. The starting point in using this framework in the Commission’s work was to examine ‘differential health outcomes’ by markers of social and economic status (such as by gender, ethnicity, education, and occupation), and then to look upstream to investigate where these differences originated. After analyzing the determinants in this way, contributors to this work of the WHO Commission then examined potential interventions to address the determinants, and suggested what needed to be measured to assess the success of those interventions (the ‘intervene’ and ‘measure’ dimensions).

Systematically reviewing the literature across the five levels and three dimensions is beyond the scope of a single review. Our aim is to provide a solid foundation for further work on health inequities in diabetes in the Caribbean by reviewing the social distribution of diabetes (type 1 and type 2); the social distribution of the risk factors (vulnerabilities); and the social distribution of its major complications, including diabetes related mortality (consequences).

**Research Questions**

**Main research question:** What is the distribution by known social determinants of health of diabetes, its risk factors and its adverse outcomes in populations living in the Caribbean?

**Secondary research question:** What are the implications of this distribution for reducing and avoiding further health inequities within the Caribbean region?

**Figure -** WHO Commission on Social Determinants Analytical Framework for Priority Public Health Conditions (2)

**Methods**

**Scoping exercise**

Before agreeing on the main research question described above, a scoping exercise was carried out in Pubmed and Embase, of the existing literature related to social determinants of diabetes within the Caribbean region. A scoping review is designed to map the existing literature, and can be used to determine what type of systematic review research questions are answerable given the currently published literature. Literature over the past 20 years was searched using an agreed search strategy to include a broad list of search terms with the following limits: English, Dutch, Spanish and French; Humans; studies done between January 1^st^, 1992 and Dec 31^st^, 2011***.*** From the list generated by the search, relevant articles were identified and grouped based on relevance after reviewing the title and/or abstract given in Pubmed or Embase.

The citations identified by the scoping exercise were downloaded into a reference manager (Endnote) and the titles and abstracts independently reviewed by two individuals. Citations were examined for relevance against the criteria described below. When it was not possible to make a decision based on the title and abstract the full text of the article was retrieved. Citations were only excluded when both reviewers agreed that they were not relevant. Where doubt existed the citation was retained and the full text sought in order to decide on the relevance of the article.

This scoping exercise established that the above systematic review primary research question was likely to be answerable from the existing literature. The scoping review demonstrated that most relevant literature existed for the English speaking Caribbean, and that less literature existed for the Dutch, French and Spanish speaking Caribbean.

**Search terms**

The search terms that were used for the scoping exercise and will be used in the full review are given in the appendix. These terms will be used in Pubmed, Embase (through Ovid), and in LILACS, MedCarib, IBECS, PAHO and WHOLIS databases (through the “Virtual Health Library). The search will be limited to articles about humans. Initially no language limits will be applied (in order to identify all relevant publications irrespective of language), followed by limiting to Dutch, English, French and Spanish. The publication dates for the full review are 1^st^ January 1992 to December 31^st^ 2012.The search terms for the social determinants were guided by the extension of the PRISMA statement for reporting systematic reviews with a focus on health equity (PLoS Medicine 2012;9:e1001333). The statement recommends using the PROGRESS-Plus checklist i.e. place of residence, race/ethnicity/culture/language, occupation, gender/sex, religion, education, socio-economic status, social capital and “plus” to indicate other possible factors.

**Selection of articles for data abstraction**

The same process of shifting through citations for the scoping review will be used for the main review – with all of them being appraised independently by two reviewers. The full text of *all articles identified as potentially relevant* will be examined. Articles will only be excluded when both reviewers agree that they are not relevant i.e. where doubt exists they will be included. Their content will be evaluated against the following inclusion/exclusion criteria. If they meet the inclusion/exclusion criteria given below detailed data abstraction will be undertaken.

Inclusion Criteria:

- Persons living in the Caribbean region
- Observational and interventional studies
- Sample size > or = 50
- Age of study participants: In reviewing diabetes outcomes, only papers describing studies done in adults (18 and older) will be abstracted while for the risk factors (obesity, physical inactivity, poor diet and smoking) studies examining persons 12 years and older will be included.
- The study describes the distribution of one or more factors in (1) or (2) in the table below by one or more social factors in (3).

Exclusion Criteria:

- Narrative review papers, commentaries, case series, qualitative studies and single case reports will notbe included. Literature on Caribbean diaspora (as opposed to populations living within the Caribbean) will notbe included.
- Sample size <50. It is expected that studies with a small sample size will be less likely than larger studies to be representative of the population.

*While case series and qualitative papers are excluded for purposes of data extraction, information from these types of studies will be used to inform the discussion.

**Table 1: Key variables to be abstracted collected**

| Group |  |  |
| --- | --- | --- |
| **1** | Outcomes  **Age:18 and up** | Incidence, prevalence, complications- diabetes mortality, glucose control (based on HBA1c and/or fasting glucose), amputations, nephropathy, neuropathy, retinopathy, blood pressure control |
| **2** | Risk factor  **Age: 12 and up** | Obesity, Physical activity/inactivity, Poor diet, smoking, metabolic syndrome |
| **3** | Social distribution | Race/ethnicity, gender, education, occupation, income/wealth |

**Data Abstraction**

A data abstraction form has been created in Microsoft Access in order to manage the data.Each full text paper will be independently data abstracted by two reviewers. Differences in data abstraction will be resolved meeting between the two reviewers reaching consensus through meeting. The data abstraction form has been designed to extract key study characteristics and findings relevant to the primary research question. In addition, it has been designed to enable an assessment to be made of the quality or risk of bias in the study. The content of the data abstraction form has been guided by the STROBE statement (5), on the reporting observational epidemiology, and by the PRISMA statement on systematic reviews concerning health equity (6).

**Quality and Risk of Bias Assessment**

The following pragmatic criteria will be used to assess the quality of the study and risk of bias for individual analyses of the social distribution of a risk factor or outcome. As for other aspects of data abstraction, data on these criteria will be abstracted by two independent reviewers, and where they disagree they will meet to reach consensus. These criteria have been designed for cross sectional and cohort, as it was found in the scoping review that the vast majority of relevant studies are of this type. The need for criteria for case control studies and trials will be reviewed if relevant studies with these designs are discovered.

***High Quality, Low Risk of Bias***

For an analysis to be classified as ‘high quality, low risk of bias’, **all** of the following criteria must be met:

The ***study*** must be:

- Be population based
- If cross sectional, sample size > 500, response rate > 75%
- If cohort study, study population representative of target population, attrition rate over the reported study period <25%, and > 100 outcome events of interest.

The ***analysis*** of social distribution must:

- Be Based on an objective measure of the risk factor/outcome
- Be Based on transparent, justified and locally appropriate approach to assessing the social factor
- Include adjustment for age and any other potential confounders, or satisfactorily demonstrate why adjustment was not required
- Exclude less than 15% of potentially eligible individuals because of missing data

*Medium Quality, Medium Risk of Bias*

For an analysis to be classified as ‘medium quality, medium risk of bias’, the following criteria must be met:

The ***study*** must be:

- Be population based
- If cross sectional, sample size of at least 250 **and/or** response rate of at least >50%
- If cohort study, study population representative of target population, attrition rate over the reported study period of <50%, **and/or**>50 outcome events of interest

The ***analysis*** of the social distribution must:

- Be based on a subjective measure (e.g. questionnaire) of the risk factor/outcome
- Be based on transparent, justified and locally appropriate approach to assessing the social factor
- Include adjustment for age and any other potential confounders, or satisfactorily demonstrate why adjustment was not required
- Exclude less than 10% of potentially eligible individuals because of missing data

*Low or Unclear Quality, High Risk of Bias*

All analyses that do not fit into high or medium categories will fall into this category. They will be described as ‘low quality’ when enough detail is available in the paper to indicate **one or more** of the following:

- Not population based
- Inappropriate/unjustified approach to assessing social factors
- Inappropriate/not justified analysis of the distribution of risk factors/outcomes by social factors, such as lack of adjustment important confounders, such as age.
- If cross sectional study sample size < 250 **and/or** response rate < 50%
- If a cohort study, attrition >50% **and/or** number of events <50
- Exclude 15% or more potentially eligible individuals for the analysis because of missing data

Studies that do not meet the criteria for ‘low quality’ will be classified as ‘quality unclear’ if it cannot be determined from what is presented in the paper whether or not the study meets **any one** of the criteria listed under ‘medium’ or ‘high’.

**Data Synthesis**

The studies will be systematically reviewed qualitatively. Given the anticipated marked heterogeneity of the studies that will be identified and abstracted it is unlikely that data synthesis i.e. meta-analyses will be conducted – however this will be kept under review. It is most likely that a narrative synthesis will be undertaken given the diversity in outcomes and potential subjectivity of some of the social determinants to be reviewed.

**Dissemination**

It is expected that the findings from the systematic review will be submitted for publication in a leading international public health journal. In addition, findings will be shared at Caribbean regional meetings such as the Caribbean Health Research Council’s annual meeting.

**References**

1.United Nations General Assembly 66th session (2011). A/RES/66/2 Political Declaration of the High-level Meeting of the General Assembly on the Prevention and Control of Non-communicable Diseases.

2.Blas, E., A. S. Kurup, et al. (2010). Equity, Social Determinants and Public Health Programmes, World Health Organization.

3. Ferguson TS, Tulloch-Reid MK, Wilks RJ. (2010). *The epidemiology of diabetes mellitus in Jamaica and the Caribbean: a historical review*. West Indian Med J. 59(3):259-64.

4. Soyibo AK, Roberts L, Barton EN (2011). *Chronic kidney disease in the Caribbean*. West Indian Med J. 60(4):464-70.

5.von Elm E, Altman DG, Egger M, Pocock SJ, Gøtzsche PC, Vandenbroucke JP; STROBE Initiative (2008). The Strengthening the Reporting of Observational Studies in Epidemiology (STROBE)statement: guidelines for reporting observational studies. J Clin Epidemiol. 61(4):344-9.

6.Welch V, Petticrew M, Tugwell P, Moher D, O'Neill J, et al. (2012) PRISMA-Equity 2012 Extension: Reporting Guidelines for Systematic Reviews with a Focus on Health Equity. PLoS Med 9(10).

**Appendix**

**Search Terms and Search Strategy for Pubmed**

1. **Social determinants**

"Health Status Disparities"[Mesh] OR "Socioeconomic Factors"[Mesh] OR "Vulnerable Populations"[Mesh] OR "Sociology, Medical"[Mesh] OR "Prejudice"[Mesh] OR "Insurance, Health"[Mesh] OR "Health Services"[Mesh] OR "Continental Population Groups"[Mesh] OR "Ethnic Groups"[Mesh] OR "Social Conditions"[Mesh] OR "Urban Health"[Mesh] OR "Urban Population"[Mesh] OR "Rural Population"[Mesh] OR Socio*[tw] OR social position[tw] OR educat*[tw] OR gender[tw] OR ethnic*[tw] OR race[tw] OR poverty[tw] OR social determinant*[tw] OR social support[tw] OR social capital[tw] OR (religion[tw] AND discrimination[tw]) OR different*[tw] OR culture[tw]OR occupation*[tw] OR income*[tw]

1. **Diabetes, risk factors and complications**

"Nutritional and Metabolic Diseases"[Mesh] OR "Diabetes Mellitus"[Mesh] OR "Glucose Intolerance"[Mesh] OR "Prediabetic State"[Mesh] OR Diabet*[tw] OR sugar[tw] OR impaired glucose tolerance[tw] OR IGT[tw] OR impaired fasting glucose[tw] OR prediabetes[tw] OR IFG[tw] OR glycem*[tw] OR glycaem*[tw] OR borderline diabet*[tw] OR Oral glucose tolerance test[tw] OR OGTT[tw] OR glucose[tw] OR hba*[tw] OR glycated[tw] OR retinopathy[tw] OR nephropathy[tw] OR neuropathy[tw] OR diabetic foot[tw] OR metabolic syndrome[tw] OR insulin resistance[tw] OR insulin sensitivity[tw] OR insulin insensitivity[tw] OR syndrome X[tw] OR Body mass index[tw] OR bmi[tw] OR waist[tw] OR waist circumference[tw] OR abdominal[tw] OR obesity[tw] OR physical activity[tw] OR exercise[tw] OR physical inactivity[tw] OR alcohol[tw] OR smoke[tw] OR smoking[tw] OR diet[tw] OR cardiometabolic[tw] OR cardio metabolic[tw] OR cardio-metabolic[tw]

1. **Caribbean region and countries^1^**

"Caribbean Region"[Mesh] OR Anguilla OR Antigua OR Bahamas OR Barbuda OR Barbados OR Belize OR Bermuda OR Cayman OR Dominica OR Grenada OR Guyana OR Haiti OR Jamaica OR Montserrat OR Kitts OR Nevis OR Lucia OR Vincent OR Grenadines OR Suriname OR Trinidad OR Tobago OR Turks OR Caicos OR Virgin Islands OR Aruba OR Antilles OR Cuba OR Puerto OR Guadeloupe OR Martinique

**Combinations and limits**

1 AND 2 AND 3, limited to humans and dates 1^st^ Jan 1992 to 31^st^ December 2012

**Search Terms and Strategy for EMBASE (through OVID) and Virtual Health Library (VHL) Selected Databases**

The same terms and strategy will be used for EMBASE, and for searching LILACS, MedCarib, IBECS, PAHO and WHOLIS through the VHL. However, for these searches MESH headings will not be specified, and all terms will be entered as text, meaning that all fields will be searched. Finally it should be noted that for each search the appropriate truncation symbol was used e.g. * for Pubmed and Ovid (note Ovid uses * or $), $ for VHL.
