# Supplementary material for: Female Gender Is a Social Determinant of Diabetes in the Caribbean: A Systematic Review and Meta-Analysis
Source: PLoS One. 2015 May 21;10(5):e0126799. doi: 10.1371/journal.pone.0126799 (PMC4440736; doi:10.1371/journal.pone.0126799)
Supplement: S1 Table — Only cross-sectional studies have been included. (DOCX) [file pone.0126799.s003.docx]

Table S1. Studies examining gender and physical activity.

| **Author, year published** | **Sample size** | **Country** | **Age range** | **Statement of results** | **Risk of bias assessment** |
| --- | --- | --- | --- | --- | --- |
| Ageyamang, 2009 | 855 | Suriname | 12-17 | The proportion (%) of **boys/girls** performing exercise >5-7dys/week by ethnicity are as follows: Hindustani=18.6/7.4; Creole=28.2/6.0; Javanese=20.0/4.4; Maroon=43.9/4.0; Mixed=29.1/8.2. | School-based  Response rate: 100% (no missing data). Adjusted for ethnicity. The statistical assessment comparing figures for boys versus girls. PA measured by questionnaire. |
| Atallah, 2007 | 2420 | Guadeloupe | 18-69 | A higher proportion of females were classified as sedentary compared to males. (70.5% versus 83.3%) | Health facility-centre based  PA assessed by questionnaire. No adjustment for confounders. Convenience sample. No statement of response rate |
| Atallah, 2011 | 685 | Guadeloupe | 18-74 | Performing moderate PA 5 times per week, was done by 67.5% of men vs 51.2% of women. While vigorous activity at least 5 days/week was done by 40.5% vs 21.6 % of men and women respectively p<0.001 | Population-based  PA measured using IPAQ questionnaire. Response rate 50%. No adjustment for confounders. |
| Barbosa, 2011 | 3413 | Barbados and Cuba | 60+ and older | In Barbados 52.8% of males and 60.0% of females were physically inactive. p=0.007 In Cuba, the proportion of those physically inactive were higher 69.9 and 81.5% p-0.001for males and females resp. | Population-based  Response rate 84.5% in Barbados and 95.3% in Cuba. PA measured by non validated questionnaire. (3 or more times per week in the past 12 months) |
| Block, 2012 | 2017 | Grenada | 18 and older (18-104) | Percentages of leisure time spent sedentary for >10min/d for men by age group <35 = 72.8, 35-44=80.9, 45-54=79.7, 55-64 = 80.7, >=65 =93.7.  For women by age group: <35 = 78.1, 35-44=79.9, 45-54=82.9, 55-64 = 83.8, >=65 =83.5. | Population-based  64% response rate; subjectively measured using a modified version of the WHO STEPS survey |
| Cumberbatch, 2011 | 2432 | Jamaica | 15-74 | Amongst men, levels(%) of high, medium and low PA are 47.2, 22.9, 29.9 respectively while amongst women the levels were 18.8, 19.2, 62.0. These sex differences were statistically significant. p<0.01 | Population-based  Lack of age adjustment (although mean ages of men and women virtually identical), and underlying issue with the sample (twice as many women) |
| Ferguson, 2008 | 2012 | Jamaica | 15-74 | The proportion of men with low levels of PA was 21.3% (95% CI),17.3–25.4 and 50.9% 95% CI (46.8–54.9) for women. | Population-based  Analysis restricted to 1972 (of 2012 participants) with blood pressure data; only crude prevalence of RF; subjectively measured; response rate not reported in this paper (referred to other paper); but population based, sample size <500. |
| Ferguson, 2010 | 839 | Jamaica | 18-20 | Proportion % (SE): Low: M18.0 (2.0) F47.1 (2.3) Total34.0 (1.6) (p < 0.001). Moderate: M45.2 (2.6) F39.7 (2.3) T42.3 (1.7) (not sign). High M36.8 (2.5) F13.2 (1.6) T23.8 (1.5) (p < 0.001) | Population-based  Subjectively measured (only assessing leisure time!); population based; Response rate > 50%. No age adjustment. |
| Ferguson, 2011 | 2848 | Jamaica | 15-74 | Levels of “Inactivity” were 16.0% and 43.0% for men and women respectively (p <0.001).  The proportions of men and women with low PA were 12.2% and 19.2% (p < 0.05);  those with high PA were males 48.0% and females 18.2% (p <0.001) | Population-based  UNCLEAR not age adjusted, missing data unclear (data weighted for missing values), subjectively measured, |
| Modeste, 2007 | 407 | Barbados | 18-74 | 16.2% versus 34.3% of men and women respectively reported having no regular vigorous weekly exercise or exercised less than once per week | Faith-based study  Not population-based. No statistical assessment of random error. |
| Rodrigues Barbosa, 2010 | 1508 | Barbados | 60+ and older | Male PA levels were 46.7% compared to females PA which was 39.9%. This difference was significant at the 5% level of testing | Population-based  PA measured by questionnaire |
| Sinnapah, 2009a | 122 | Guadeloupe | 17-66 | For both Asian Indians and other races, females had a higher level of physical activity.* than males. These differences were statistically significant (P<0.05 in both instances. | Health facility- based  PA measured by questionnaire. Stratified by ethnicity |
| Sinnapah, 2009c | 780 | Guadeloupe | 10-18 | In both Asian Indians and controls, females reported lower leisure time physical activity compared to their male counterparts. (Mean LTPA* Asian Indians: Males/females 1.54+/-1.62 / 1.25+/-1.19 Controls: Males/Females 1.67+/-1.43 1.51+/-1.29 | School-based  Low/Medium. PA measured by self-report via questionnaire. School-based sample, 780 participants. Response rate (83%). RF measured by questionnaire. There was age matching. |

***LTPA= Leisure time Physical Activity**

**PA = Physical Activity**
