# Supplementary material for: Female Gender Is a Social Determinant of Diabetes in the Caribbean: A Systematic Review and Meta-Analysis
Source: PLoS One. 2015 May 21;10(5):e0126799. doi: 10.1371/journal.pone.0126799 (PMC4440736; doi:10.1371/journal.pone.0126799)
Supplement: S2 Table — Mean body mass index has been used as the measure of obesity. (DOCX) [file pone.0126799.s004.docx]

Table S2: Studies examining obesity by gender

| **Author, year published** | **Sample size** | **Country** | **Age range** | **Statement of results(Mean BMI)** | | **Risk of bias assessment** |
| --- | --- | --- | --- | --- | --- | --- |
| Agyemang, 2009 | 855 | Suriname | 12-17 | Males | Females | School-based  Response rate: 100%.  BMI objectively measured. Stratified by ethnicity; no further adjustment for confounders. |
|  |  |  |  | Hindustani =20.1 Creole=21.0  Javanese=20.2 Maroon=19.3 Mixed=20.7  BMI by ethnicity trends: males: p=0.18; | Hindustani= 19.5  Creole= 20.8  Javanese= 19.3  Maroon= 21.3  Mixed= 20.3  BMI by ethnicity trends: females: p=0.02 |  |
| Anderson 2011 | 857 | Jamaica | 24-74 | 23.5kg/m2 (95% CI, 23.1 - 23.9) | 27.6 kg/m2 ( 95% CI, 27.1-28.1) | Population-based  Response rate: 62%;  BMI objectively measured; No adjustment for age was necessary. |
| Barcelo, 2007 | 13753 | Cuba, Barbados(SABE) | >60 | Barbados:25.4(0.5)  Cuba: 23.2(0.2) | Barbados: 28.1(0.3)  Cuba: 25.6(0.2) | Population-based  Response rate: 81% in Barbados, 95.3% in Cuba  BMI objectively measured. |
| Block, 2012 | 2017 | Grenada | 18-104 | By age group  <35 25.4  35-44 27.2  45-54 26.6  55-64 26.6  >=65 25.8  P=05. | By age group  <35 27.7  35-44 29.9  45-54 30.7  55-64 29.8  >=65 28.9  p<.001 | Population-based  Response rate: 64%  BMI objectively measured |
| Boyne, 2010 | 393 | Jamaica | 25-74 | 25.0 (SD 4.6) | 29.7 (SD 6.5) | Population-based  Cohort study  Attrition rate<50% |
| Cumberbatch, 2011 | 2432 | Jamaica | 15-74 | 24.7( SD 5.4) | 28.9 (SD 6.8), p<0.01 | Population-based  Response rate: 98% |
| da Silva Coqueiro, 2009 | 1905 | Cuba | >60 | 60-64: 24.4  65-69: 23.3  70-74: 22.7  75-79: 22.2  80+: 22.2 | 27.2  26.6  25.2  24.5  23.6 | Population-based  Response rate: 95.3% |
| Ferguson, 2010a | 708 | Jamaica | 25-74 | 23.3 (SD 4.0); | 27.6 (SD 6.4); | Population- based  Objective measures,  No missing data for this relationship;  Lack of adjustment for confounders |
| Ferguson, 2010b | 839 | Jamaica | 18-20 | 22.73 (SD 4.37) | 23.35 (SD 5.60) | Population-based; BMI Objectively measured; No age adjustment |
| Foucan, 2007 | 966 | Guadeloupe | 18-74 | Mean (IQR): 24 (21-27) | Mean (IQR) 26 (22-29) | Health-facility-based; No missing values; Objective measures; no response rate reported, no age adjustment |
| Nam, 2012 | 5786 | Cuba, Barbados | >65 | Stratified by Low and High Waist Circumference: Mean BMI (SE) Bridgetown:  LWC 25.0(0.3) HWC 31.4(0.9),  Havana:  LWC 23.1(0.2) HWC 28.8(0.5); | Stratified by Low and High Waist Circumference: Mean BMI (SE) Bridgetown:  LWC 24.1(0.3) HWC 31.2(0.4),  Havana:  LWC 23.2(0.2) HWC28.9(0.4); | Population-based; Response rate: 81% in Barbados, 95.3% in Cuba; BMI objectively measured; BMI reported by gender, age and education, weighted |
| Sinnapah, 2009a | 12 | Guadeloupe | 17-66 | Asian Indian –  25.2 ± 3.4  Control Ethnicity- 25.6 ± 3.6 | Asian Indian-  24.4 ± 4.0  Control ethnicity - 24.4 ± 4.3 | Health facility-based; Adjusted for ethnicity only |
| Sinnapah 2009b | 720 | Guadeloupe | 11-17 | Age<14  Asian Indian  19.6 ± 4.0  Control group  19.3±3.7  Age>14  Asian Indian  21.0 ± 4.7  Control group  20.8 ±3.8 | Age<14  Asian Indian  19.0 ± 3.5  Control group  20.3± 4.0  Age>14  Asian Indian  21.1 ± 5.3  Control group  21.4 ± 4.0 | School-based; Response rate: 78%; BMI measured objectively. |
| Sinnapah 2009c | 780 | Guadeloupe | 10-18 | Asian Indian  19.6 ± 4.2  Control group  19.9 ± 3.6 | Asian Indian  18.8 ± 3.0  Control group  20.2 ± 3.7 | School-based; Response rate: 83%; Source of BMI unclear. |
| Soloway, 2009 | 278 | Saba | >18 years | 27.8 | 28.7 | Population-based; Response rate: 68%; BMI objectively measured |
