# Supplementary material for: Female Gender Is a Social Determinant of Diabetes in the Caribbean: A Systematic Review and Meta-Analysis
Source: PLoS One. 2015 May 21;10(5):e0126799. doi: 10.1371/journal.pone.0126799 (PMC4440736; doi:10.1371/journal.pone.0126799)
Supplement: S3 Table — (DOCX) [file pone.0126799.s005.docx]

Table S3: Characteristics of studies describing the relationship between diabetes control and gender

| **Author, year published** | **No. of males/N** | **Country** | **Age range** | **Measure of control** | **Results** | **Risk of bias assessment** |
| --- | --- | --- | --- | --- | --- | --- |
| Apparico, 2007 | 39/132 | Trinidad | 27-81 | HbA1C > 7.0% = poor control | % Poorly controlled: Males 46.2%; Females 58.1% p=0.171 | Health facility-based; No adjustment for potential confounders |
| Ferguson, 2011 | 883/2848 | Jamaica | 15-74 | Fasting glucose | % Uncontrolled: Males 49.9 Females 54.3 | Population-based; not age adjusted, missing data unclear (data weighted for missing values), |
| Nunez, 2011 | 8/53 | US Virgin Island | 26-80 | HbA1C | Men had significantly tighter glycaemic control than women p=0.0271 | Health facility-based; 'Purposive' sample, claims assessment of association of HBA1c with various factors but shows no data for this. |
